# The effect of cigarette modified risk claims and brand on perceived risk, product appeal, and use intentions

**DOI:** 10.1371/journal.pone.0274097

**Published:** 2022-10-03

**Authors:** Erin Keely O’Brien, Andrea L. Ruybal, Amber R. . Koblitz, Sarah E. Johnson

**Affiliations:** Office of Science, Center for Tobacco Products, Food and Drug Administration (FDA), Silver Spring, MD, United States of America; George Washington University, UNITED STATES

## Abstract

**Objectives:**

No studies have examined the brand context in which modified risk claims appear on tobacco products. This study examines how marketing products with modified risk claims affects risk perceptions, appeal, and intentions among own-brand, other brand, and novel brand cigarettes.

**Methods:**

This experiment employed a 3 (claim: risk modification [RM], exposure modification [EM], control) x 3 (brand: own, other, novel) between-subjects design. A convenience sample (N = 1,557, M_age_ = 40.28, SD_age_ = 19.01, 71.3% female, 80.3% White) of current or former Marlboro, Camel, or Newport users was collected. Participants were assigned to view their own brand, another brand, or a novel brand, with or without a claim, and rated perceived risk after switching to this product, product appeal, and use intentions.

**Results:**

Participants in the RM or EM conditions had lower risk perceptions (versus control). Claim did not affect appeal. Adult established cigarette users in the EM (but not RM) condition had higher intentions (versus control). Participants rated their own and another brand as more appealing than the novel brand. Interactions between brand and claim were not significant.

**Conclusions:**

We found modified risk claims decreased risk perceptions but did not impact appeal. Whereas participants showed preference for their own brand in terms of appeal and intentions, brand did not moderate the impact of claims.

## Introduction

Historically, tobacco companies have marketed products as lower risk when they were not, in fact, lower risk [1]. To legally market a tobacco product in the U.S. as lower risk than other products (modified risk) or as presenting a lower risk of exposure to a harmful substance (modified exposure), tobacco companies must submit applications for the Food and Drug Administration (FDA) to evaluate [2]. To meet the standards for authorization, FDA must determine, among other things, that the marketing of the product will benefit the U.S. population as a whole [3]. As part of demonstrating this, companies can submit studies of how consumers perceive and intend to use products marketed with modified risk claims [4, 5].

Research has described how consumer perceptions and intentions change in response to modified risk information [6–10]. These studies typically found that including modified risk claims reduces risk perception of the product among cigarette users and nonusers, and can increase purchase intentions among cigarette users. All such studies examined the effect of claims on smokeless tobacco, and one included e-cigarettes [8]. Research on cigarette modified risk claims has been limited to claims about nicotine content, and found that cigarettes marketed as low nicotine are perceived as having lower health and addiction risks [11, 12]. To our knowledge, no studies have examined the role of brand in how consumers respond to modified risk information. It is possible that consumers are particularly responsive to modified risk information appearing on their own brand, compared to another known or unknown brand, because of positive brand relationships [13]. For instance, consumer research finds that familiar brands may be perceived as more credible [14], and claims these brands make may be more impactful [15].

The current study examines, in an online convenience sample, how risk perceptions, product appeal, and intention to switch to the product are impacted by (a) viewing modified risk or modified exposure claims (compared to no claim), and (b) viewing one’s own brand or another leading brand (compared to a novel brand).

## Method

This experiment employed a 3 (claim type: risk modification [RM], exposure modification [EM], control condition) x 3 (brand type: own, other, novel) between-subjects factorial design.

### Sample

Participants were recruited from the Lightspeed Research online consumer panel from May-June 2017. This study included four age and cigarette smoking status subgroups relevant to consider in modified risk tobacco product applications [4, 5]: young adult experimental cigarette users, young adult established cigarette users, adult established cigarette users, and adult former cigarette users (N = 1,557). Established cigarette users had smoked at least 100 cigarettes and smoked every day or some days. Experimental cigarette users had smoked fewer than 100 cigarettes and smoked some days. Former cigarette users completely quit 1+ years ago and had smoked at least 100 cigarettes. Participants’ age ranged from 18 to 89 years (M = 40.28, SD = 19.01). Participants aged 18–24 were classified as young adults, while participants aged 25 and above were classified as adults. This categorization of young adults and adults is common in tobacco research [16, 17] as young adults are more likely to initiate tobacco use and more likely to use multiple products compared to older adults [18, 19]. Most participants identified as female (71.3%), White (80.3%), and reported household incomes under $60,000 (66.6%). See Table 1 for participant demographic information by cigarette use status. All participants were current or past cigarette users of one of three brands (Marlboro, Camel, or Newport), to facilitate brand condition assignment as described below.

**Table 1:** Descriptive statistics by sample.

	Young adult established cigarette users N = 451	Young adult experimental cigarette users N = 167	Adult established cigarette users N = 475	Adult former cigarette users N = 464
**Age** M (SD)	21.77 (1.85)	20.60 (1.92)	47.93 (12.24)	57.52 (15.00)
**Sex**				
Male	14.2%	22.8%	25.7%	45.9%
Female	85.4%	76.6%	72.8%	54.1%
Missing	0.4%	0.6%	1.5%	0.0%
**Race/ethnicity**				
Hispanic*	2.9%	10.2%	2.1%	0.2%
Black/African American	8.6%	15.7%	7.6%	4.3%
White	77.6%	55.7%	81.9%	90.1%
Asian American	2.9%	4.2%	2.5%	0.9%
Other	4.9%	11.4%	2.7%	3.7%
Missing	3.1%	3.0%	3.2%	0.9%
**Income**				
$0-$24,999	46.1%	43.1%	26.3%	18.3%
$25,000-$59,999	32.4%	24.0%	38.5%	38.6%
$60,000+	11.3%	21.0%	28.6%	38.6%
Missing	10.2%	12.0%	6.5%	4.5%

Note.

*Ethnicity was assessed in a separate question from race; for analysis, all respondents affirming Hispanic ethnicity were counted in this category, and other categories represent those identifying as non-Hispanic.

### Procedure and measures

Participants reported their preferred brand of cigarettes and were randomly assigned to view a cigarette pack or ad for a new variety of their own brand (Marlboro, Camel, or Newport “Select”), another of these brands, or a novel brand (“Durham,” created for this study). They were randomized to see an ad or pack that either contained no claim, the RM claim, “Lower risk of heart disease than other cigarettes”, or the EM claim, “25% fewer nitrosamines than other cigarettes”. See Table 2 for sample sizes by claim and brand condition.

**Table 2:** Sample size by claim and brand condition.

	Young adult established cigarette users	Young adult experimental cigarette users	Adult established cigarette users	Adult former cigarette users
Total N	N = 451	N = 167	N = 475	N = 464
**Claim Condition**				
Control	n = 150	n = 56	n = 157	n = 154
Risk Modification	n = 151	n = 54	n = 159	n = 155
Exposure Modification	n = 150	n = 57	n = 159	n = 155
**Brand Condition**				
Own	n = 150	n = 56	n = 158	n = 156
Other	n = 150	n = 55	n = 160	n = 154
Novel	n = 151	n = 56	n = 157	n = 154

Participants (except for former cigarette users) then responded to six items [20–24] adapted to ask them to imagine they had completely switched to the new cigarettes and rate their risk of getting different diseases (referred to as “perceived risk,” α = .88). All participants responded to nine adapted [25] items about the appeal of the product (α = .77) and three adapted [20] items (α = .92) assessing intention to use the product. Participants were debriefed regarding the hypothetical nature of the products and claims, asked to confirm their understanding—with an affirmative response—that the stimuli were made up for the purposes of the study. The study protocol was approved by human subjects review boards at the Research Triangle Institute and FDA and APA guidelines were followed in conducting this research.

## Results

We used an SPSS Statistics Subscription to conduct three-way Analyses of Variance to examine the effect claim and brand condition had on the outcomes for: young adult experimental cigarette users, young adult established cigarette users, adult established cigarette users, and adult former cigarette users (Table 2). Post-hoc tests were Tukey honestly significant difference tests.

Claim condition had a main effect on perceived risk for all age and smoking status subgroups (Table 3). Perceived risk was lower in both the RM and EM conditions compared to the control; and perceived risk in the RM and EM conditions did not significantly differ. Claim condition did not have a main effect on product appeal, and only had an effect on purchase intentions among adult established cigarette users. For this subgroup, perceived risk was lower in the EM condition compared to the control condition and did not differ between other claim conditions.

**Table 3:** Effects of claim and brand condition on perceived risk, product appeal, and intentions to try/purchase.

Factor	Young adult estab. cigarette users	Young adult exper. cigarette users	Adult estab. cigarette users	Adult former cigarette users
	*F(df)*, *pη*^*2*^	*F(df)*, *pη*^*2*^	*F(df)*, *pη*^*2*^	*F(df)*, *pη*^*2*^
	M (SE), 95% CI	M (SE), 95% CI	M (SE), 95% CI	M (SE), 95% CI
Outcome: Perceived Risk^1^				
	*R*^*2*^ = .04	*R*^*2*^ = .14	*R*^*2*^ = .06	-
Claim^2^	*F(2*, *442) = 6*.*58*,** *pη*^*2*^ *=* .*03*	*F(2*, *158) = 7*.*94*,*** *pη*^*2*^ *=* .*09*	*F(2*, *466) = 10*.*01*,*** *pη*^*2*^ *=* .*04*	-
Control	3.05 (.04), [2.97, 3.13]	3.20 (.08), [3.04, 3.36]	3.03 (.03), [2.97, 3.09]	-
Risk Modification	2.85 (.03), [2.78, 2.91]	2.75 (.08), [2.59, 2.90]	2.87 (.03), [2.81, 2.94]	-
Exposure Modification	2.91 (.04), [2.82, 3.00]	2.92 (.09), [2.75, 3.10]	2.84 (.03), [2.77, 2.91]	-
Brand	*F(2*, *442) = 2*.*28*, *pη*^*2*^ *=* .*01*	*F(2*, *158) = 3*.*30*, *pη*^*2*^ *=* .*04*	*F(2*, *466) = 0*.*29*, *pη*^*2*^ *=* .*00*	-
Own	3.00 (.04), [2.91, 3.09]	2.86 (.07), [2.71, 3.00]	2.91 (.03), [2.85, 2.98]	-
Other	2.93 (.03), [2.86, 3.00]	3.13 (.09), [2.95, 3.30]	2.93 (.03), [2.86, 3.00]	-
Novel	2.88 (.04), [2.79, 2.96]	2.90 (.09), [2.71, 3.09]	2.90 (.03), [2.83, 2.96]	-
Claim * Brand	*F(4*, *442) = 0*.*65*, *pη*^*2*^ *=* .*01*	*F(4*, *158) = 0*.*87*, *pη*^*2*^ *=* .*02*	*F(4*, *466) = 2*.*27*, *pη*^*2*^ *=* .*02*	-
Outcome: Product Appeal^3^				
	*R*^*2*^ = .08	*R*^*2*^ = .10	*R*^*2*^ = .06	*R*^*2*^ = .03
Claim	*F(2*, *442) = 0*.*79*, *pη*^*2*^ *=* .*00*	*F(2*, *158) = 2*.*18*, *pη*^*2*^ *=* .*03*	*F(2*, *466) = 0*.*17*, *pη*^*2*^ *=* .*00*	*F(2*, *455) = 2*.*01*, *pη*^*2*^ *=* .*01*
Control	2.95 (.05), [2.85, 3.05]	3.05 (.08), [2.89, 3.21]	3.09 (.05), [3.00, 3.18]	2.77 (.05), [2.67, 2.87]
Risk Modification	2.99 (.05), [2.90, 3.07]	3.13 (.07), [2.99, 3.28]	3.09 (.04), [3.00, 3.17]	2.82 (.05), [2.73, 2.92]
Exposure Modification	3.03 (.05), [2.94, 3.12]	2.93 (.06), [2.81, 3.05]	3.12 (.05), [3.03, 3.21]	2.90 (.04), [2.82, 2.99]
Brand	*F(2*, *442) = 16*.*12*,*** *pη*^*2*^ *=* .*07*	*F(2*, *158) = 5*.*13*,** *pη*^*2*^ *=* .*06*	*F(2*, *466) = 13*.*95*,*** *pη*^*2*^ *=* .*06*	*F(2*, *455) = 5*.*09*,** *pη*^*2*^ *=* .*02*
Own	3.16 (.05), [3.05, 3.26]	3.20 (.06), [3.08, 3.33]	3.21 (.04), [3.13, 3.30]	2.91 (.05), [2.81, 3.01]
Other	3.02 (.05), [2.93, 3.11]	2.89 (.07), [2.76, 3.03]	3.17 (.05), [3.08, 3.26]	2.88 (.05), [2.79, 2.97]
Novel	2.79 (.04), [2.71, 2.87]	3.01 (.08), [2.85, 3.16]	2.91 (.04), [2.83, 2.99]	2.71 (.04), [2.63, 2.80]
Claim * Brand	*F(4*, *442) = 1*.*41*, *pη*^*2*^ *=* .*01*	*F(4*, *158) = 0*.*79*, *pη*^*2*^ *=* .*02*	*F(4*, *466) = 0*.*86*, *pη*^*2*^ *=* .*01*	*F(4*, *455) = 0*.*36*, *pη*^*2*^ *=* .*00*
Outcome: Intentions to Try/Purchase^4^				
	*R*^*2*^ = .03	*R*^*2*^ = .06	*R*^*2*^ = .06	*R*^*2*^ = .03
Claim	*F(2*, *442) = 2*.*44*, *pη*^*2*^ *=* .*11*	*F(2*, *158) = 0*.*63*, *pη*^*2*^ *=* .*01*	*F(2*, *466) = 5*.*08*,** *pη*^*2*^ *=* .*02*	*F(2*, *455) = 2*.*60*, *pη*^*2*^ *=* .*01*
Control	3.01 (.10), [2.82, 3.21]	2.92 (.15), [2.62, 3.23]	3.16 (.09), [2.97, 3.34]	1.36 (.06), [1.24, 1.49]
Risk Modification	3.06 (.10), [2.86, 3.25]	3.09 (.15), [2.78, 3.40]	3.34 (.09), [3.16, 3.34]	1.49 (.08), [1.34, 1.64]
Exposure Modification	3.30 (.10), [3.10, 3.49]	3.15 (.16), [2.84, 3.46]	3.57 (.09), [3.39, 3.76]	1.60 (.09), [1.44, 1.77]
Brand	*F(2*, *442) = 4*.*99*,** *pη*^*2*^ *=* .*02*	*F(2*, *158) = 3*.*47*,* *pη*^*2*^ *=* .*04*	*F(2*, *466) = 13*.*95*,*** *pη*^*2*^ *=* .*04*	*F(2*, *455) = 2*.*04*, *pη*^*2*^ *=* .*01*
Own	3.36 (.10), [3.17, 3.56]	3.30 (.15), [3.01, 3.60]	3.66 (.09), [3.49, 3.83]	1.61 (.08), [1.44, 1.77]
Other	3.06 (.10), [2.86, 3.25]	2.74 (.15), [2.43, 3.05]	3.27 (.10), [3.08, 3.46]	1.44 (.07), [1.29, 1.59]
Novel	2.94 (.09), [2.76, 3.13]	3.11 (.15), [2.81, 3.42]	3.14 (.10), [2.96, 3.33]	1.41 (.07), [1.27, 1.54]
Claim * Brand	*F(4*, *442) = 0*.*20*, *pη*^*2*^ *=* .*00*	*F(4*, *158) = 0*.*23*, *pη*^*2*^ *=* .*01*	*F(4*, *466) = 0*.*86*, *pη*^*2*^ *=* .*00*	*F(4*, *455) = 0*.*74*, *pη*^*2*^ *=* .*01*

*Notes*. Higher scores indicate a higher amount of the construct (e.g., likelihood that switching brands can result in a number of health risks.)

^1^ Six questions- Imagine that tomorrow you completely switched to **[BRAND] *SELECT*** cigarettes and stopped using your usual cigarettes. How would this affect your chances of getting each of these tobacco-related health issues? If I completely switched to using [brand] cigarettes, my chances of getting a tobacco-related disease would be: getting cancer, getting lung disease, getting heart disease, having a stroke, becoming addicted. Response options 1 (Much less likely) to 5 (Much more likely). This was not assessed among adult former cigarette users.

^2^ Claim conditions: control = no claim on the ad/pack, risk modification = “lower risk of heart disease” on ad/pack, exposure modification = “25% fewer nitrosamines” on ad/pack.

^3^Nine items- I think [brand] cigarettes would: be rich in tobacco flavor, taste of cheap tobacco, be satisfying, be of the highest quality tobacco, be harsh on the throat, taste good, be popular among cigarette users, be an expensive product, be a product I might try. Response options 1 (Strongly disagree) to 5 (Strongly agree).

^4^Three items- How likely would you be to: try [brand] cigarettes in the next 6 months?, purchase [brand] cigarettes in the next 6 months?, try [brand] cigarettes if one of your best friends offered it to you? Response options 1 (Very unlikely) to 5 (Very likely).

*p < .05

**p≤.01

***p≤.001

Brand condition did not have a main effect on perceived risk for any subgroup. However, brand condition had an effect on appeal for all subgroups. For all groups except young adult experimental cigarette users, appeal was significantly greater for both own and other brand, compared to the novel brand; appeal did not differ between own brand and other brand. For young adult experimental cigarette users, appeal was significantly higher for own brand compared to another brand. Brand condition also had an effect on intentions for all subgroups except for adult former cigarette users; these subgroups reported higher intentions to use the new variety of their own brand. Young adult established cigarette users had higher intentions to use their own brand compared to a novel brand; young adult experimental cigarette users had higher intentions to use their own brand compared to other brand; and adult established cigarette users had higher intentions to use their own brand compared to both other conditions.

Brand did not moderate the effect of claims on any of the outcomes.

## Discussion

This study assessed how modified risk claims affected risk perceptions, appeal, and intention to switch to own brand, other brand, and novel brand cigarettes among four key subgroups: young adult established cigarette users, young adult experimental cigarette users, adult established cigarette users, and adult former cigarette users. Compared to participants who viewed the no claim control cigarettes, we found that those who viewed cigarettes either with the RM or EM claims had lower perceptions of risk; however, claims had no effect on product appeal. Adult established cigarette users viewing the EM claim (but not the RM claim) had higher intentions to use those cigarettes. Our findings are consistent with findings from studies of non-cigarette tobacco products. For example, some studies also found that RM [7, 8] and EM claims [7] reduced risk perceptions among current and former cigarette users; other studies found that RM claims increased product use intentions among adult cigarette users, but not among nonusers [6–9].

Brand condition affected both product appeal and intentions to switch. Participants viewed cigarettes that were their own brand or another leading brand as more appealing than the novel brand, and they generally had higher intentions to use their own brand. This suggests that newly marketed tobacco products using an existing brand (e.g., Marlboro Heatsticks for IQOS) may be more appealing and cigarette users may be more interested in using them than an unknown or new brand. Additionally, brand did not affect the impact of claims on perceived risk, inconsistent with our hypotheses that claims made by one’s own brand were more impactful [11]. Whereas the main effects of claim and brand generally had a medium effect size, the effect sizes of the interactions between these factors were small; thus we may not have had the statistical power to assess their significance. Another possibility is that research based on marketing other consumer products may not be generalizable to tobacco products—consumers have particularly low trust in health information coming from tobacco companies in general [26, 27], and this could wash out any positive effects of brand relationship when it comes to perceptions of health information. Future research could examine this in the context of novel tobacco products (e.g., e-cigarettes) and newer brands, which may not be subject to the same expectations of low trustworthiness as traditional cigarette manufacturers.

This study has several limitations. First, our experiment was designed to examine differences between conditions rather than generate population estimates. Thus, our use of a nonrepresentative sample means that point estimates cannot be generalized; however, this does not affect the study’s internal validity. Second, all measures were self-report which may have resulted in respondent bias and a subsequent underestimation of the strength of the findings. Additionally, we studied the effects of one RM claim and one EM claim; our findings related to claims may be attributable to the particular claims we tested and may not be generalizable. Similarly, because we created just one novel brand as a control, it is not clear to what extent the brand results are generalizable, or rather reflect the impact of these particular stimuli. Future studies could include additional real and novel brands.

## Conclusion

Marketing tobacco products with modified risk claims can decrease risk perceptions and increase use intentions in some cigarette use groups. Previously, no studies had examined the brand context in which modified risk claims appear. These findings provide insight into how consumers might react to potential MRTPs, including factors—such as cigarette use status and brand—that could affect those judgments. As reactions to claims may be specific to claim wording and structure [28], future research systematically examining claim type (i.e., RM vs. EM) and claim elements may inform the extent to which such effects generalize across claims.

## References

[1] Pollay RW, Dewhirst T. The dark side of marketing seemingly “Light” cigarettes: successful images and failed fact. Tob Control. 2002;11(suppl 1):i18–i31. doi: 10.1136/tc.11.suppl_1.i18 11893811 PMC1766068

[2] United States Food and Drug Administration. Modified risk Tobacco Products. 2020. https://www.fda.gov/tobacco-products/advertising-and-promotion/modified-risk-tobacco-products. Accessed February 18, 2021.

[3] United States Food and Drug Administration. Section 911 of the Federal Food, Drug, and Cosmetic Act: Modified risk tobacco products. 2018. https://www.fda.gov/tobacco-products/rules-regulations-and-guidance/section-911-federal-food-drug-and-cosmetic-act-modified-risk-tobacco-products. Accessed February 18, 2021.

[4] United States Food and Drug Administration. Draft guidance for industry: Modified risk tobacco product applications. 2012. https://www.fda.gov/media/83300/download. Accessed February 18, 2021.

[5] United States Food and Drug Administration. Draft guidance for industry: Principles for designing and conducting tobacco product perception and intention studies. 2020.https://www.fda.gov/media/143322/download. Accessed February 18, 2021.

[6] Callery WE, Hammond D, O’Connor RJ, Fong GT. The appeal of smokeless tobacco products among young Canadian smokers. Nicotine Tob Res. 2011;13(5):373–383. 10.1093/ntr/ntr013 21357730

[7] Capella ML, Taylor CR, Kees J. Tobacco harm reduction advertising in the presence of a government‐mandated warning. J Consum Aff. 2012;46(2):235–259. doi: 10.1111/j.1745-6606.2012.01229.x

[8] El-Toukhy S, Baig SA, Jeong M, Byron MJ, Ribisl KM, Brewer NT. Impact of modified risk tobacco product claims on beliefs of US adults and adolescents. Tob Control. 2018;27(Suppl 1):s62–s69. doi: 10.1136/tobaccocontrol-2018-054315 30158212 PMC6202195

[9] Mays D, Moran MB, Levy DT, Niaura RS. The impact of health warning labels for Swedish snus advertisements on young adults’ snus perceptions and behavioral intentions. Nicotine Tob Res. 2016;18(5):1371–1375. doi: 10.1093/ntr/ntv140 26116085 PMC5942605

[10] Rodu B, Plurphanswat N, Hughes JR, Fagerström K. (2016). Associations of proposed relative-risk warning labels for snus with perceptions and behavioral intentions among tobacco users and nonusers. Nicotine Tob Res. 2016;18(15):809–816. doi: 10.1093/ntr/ntv168 26253616 PMC6280995

[11] Strasser AA, Tang KZ, Tuller MD, Cappella JN. PREP advertisement features affect smokers’ beliefs regarding potential harm. Tob Control. 2008;17(Suppl 1):i32–i38. doi: 10.1136/tc.2007.022426 18768457 PMC4047638

[12] Shadel WG, Lerman C, Cappella J, Strasser AA, Pinto A, Hornik R. Evaluating smokers’ reactions to advertising for new lower nicotine quest cigarettes. Psychol Addict Behav. 2006;20(1):80–84. doi: 10.1037/0893-164X.20.1.80 16536669

[13] Johnson SE, Coleman BN, Schmitt CL. It’s complicated: Examining smokers’ relationships with their cigarette brands. Psychol Addict Behav. 2016;30(8):887–894. doi: 10.1037/adb0000225 27831717 PMC5702884

[14] Erdem T, Swait J. Brand credibility and its role in brand choice and consideration. J Consum Res. 2004;31(1):191–199.

[15] Lee SY. When do consumers believe puffery claims? The moderating role of brand familiarity and repetition. J Promot Manag. 2014;20(2),219–239.

[16] Kasza KA, Ambrose BK, Conway KP, et al. Tobacco-Product Use by Adults and Youths in the United States in 2013 and 2014 [published correction appears in N Engl J Med. 2018 Feb 1;378(5):492]. N Engl J Med. 2017;376(4):342–353. doi: 10.1056/NEJMsa1607538 28121512 PMC5317035

[17] Johnson AL, Collins LK, Villanti AC, Pearson JL, Niaura RS. Patterns of Nicotine and Tobacco Product Use in Youth and Young Adults in the United States, 2011–2015. Nicotine Tob Res. 2018;20(suppl_1):S48–S54. doi: 10.1093/ntr/nty018 30125012 PMC6093370

[18] Fix BV, O’Connor RJ, Vogl L, et al. Patterns and correlates of polytobacco use in the United States over a decade: NSDUH 2002–2011. Addict Behav. 2014;39(4):768–781. doi: 10.1016/j.addbeh.2013.12.015 24457900 PMC3984759

[19] Barrington-Trimis JL, Braymiller JL, Unger JB, et al. Trends in the Age of Cigarette Smoking Initiation Among Young Adults in the US From 2002 to 2018. JAMA Netw Open. 2020;3(10):e2019022. doi: 10.1001/jamanetworkopen.2020.19022 33021650 PMC7539122

[20] Adkison SE, Bansal-Travers M, Smith DM, O’Connor RJ, Hyland AJ. Impact of smokeless tobacco packaging on perceptions and beliefs among youth, young adults, and adults in the U.S: findings from an internet-based cross-sectional survey. Harm Reduct J. 2014;11:2. doi: 10.1186/1477-7517-11-2 24433301 PMC3942180

[21] Borland R, Cooper J, McNeill A, O’Connor R, Cummings KM. Trends in beliefs about the harmfulness and use of stop-smoking medications and smokeless tobacco products among cigarettes smokers: findings from the ITC four-country survey. Harm Reduct J. 2011;8:21. doi: 10.1186/1477-7517-8-21 21859499 PMC3170207

[22] Callery WE, Hammond D, O’Connor RJ, Fong GT. The appeal of smokeless tobacco products among young Canadian smokers: the impact of pictorial health warnings and relative risk messages. Nicotine Tob Res. 2011;13(5):373–383. doi: 10.1093/ntr/ntr013 21357730

[23] Shiffman S, Pillitteri JL, Burton SL, Di Marino ME. Smoker and ex-smoker reactions to cigarettes claiming reduced risk. Tob Control. 2004;13(1):78–84. doi: 10.1136/tc.2003.005272 14985602 PMC1747811

[24] Weinstein ND, Marcus SE, Moser RP. Smokers’ unrealistic optimism about their risk. Tob Control. 2005;14(1):55–59. doi: 10.1136/tc.2004.008375 15735301 PMC1747991

[25] Germain D, Wakefield MA, Durkin SJ. Adolescents’ perceptions of cigarette brand image: does plain packaging make a difference? J Adolesc Health. 2010;46(4):385–392. doi: 10.1016/j.jadohealth.2009.08.009 20307829

[26] Alcalá HE, Sharif MZ, Morey BN. Misplaced trust: racial differences in use of tobacco products and trust in sources of tobacco health information. Nicotine Tob Res. 2017;19(10):1199–1208. doi: 10.1093/ntr/ntx080 28387825 PMC6580933

[27] Wright J. So, whom do we trust? Ipsos-Reid. 2003. https://www.ipsos.com/en-ca/so-whom-do-we-trust. Accessed February 18, 2021.

[28] Brewer NT, Morgan JC, Baig SA, et al. Public understanding of cigarette smoke constituents: three US surveys. Tob Control. 2016;26(5):592–599. doi: 10.1136/tobaccocontrol-2015-052897 27924009 PMC5495614

